# Tick-Pathogen Interactions and Vector Competence: Identification of Molecular Drivers for Tick-Borne Diseases

**DOI:** 10.3389/fcimb.2017.00114

**Published:** 2017-04-07

**Authors:** José de la Fuente, Sandra Antunes, Sarah Bonnet, Alejandro Cabezas-Cruz, Ana G. Domingos, Agustín Estrada-Peña, Nicholas Johnson, Katherine M. Kocan, Karen L. Mansfield, Ard M. Nijhof, Anna Papa, Nataliia Rudenko, Margarita Villar, Pilar Alberdi, Alessandra Torina, Nieves Ayllón, Marie Vancova, Maryna Golovchenko, Libor Grubhoffer, Santo Caracappa, Anthony R. Fooks, Christian Gortazar, Ryan O. M. Rego

**Affiliations:** ^1^SaBio. Instituto de Investigación en Recursos Cinegéticos CSIC-UCLM-JCCMCiudad Real, Spain; ^2^Department of Veterinary Pathobiology, Center for Veterinary Health Sciences, Oklahoma State UniversityStillwater, OK, USA; ^3^Global Health and Tropical Medicine, Instituto de Higiene e Medicina Tropical, Universidade Nova de LisboaLisboa, Portugal; ^4^UMR BIPAR INRA-ANSES-ENVAMaisons-Alfort, France; ^5^Biology Centre, Czech Academy of Sciences, Institute of ParasitologyCeske Budejovice, Czechia; ^6^Faculty of Science, University of South BohemiaČeské Budějovice, Czechia; ^7^Facultad de Veterinaria, Universidad de ZaragozaZaragoza, Spain; ^8^Animal and Plant Health AgencySurrey, UK; ^9^Faculty of Health and Medicine, University of SurreyGuildford, UK; ^10^Institute of Infection and Global Health, University of LiverpoolLiverpool, UK; ^11^Institute for Parasitology and Tropical Veterinary Medicine, Freie Universität BerlinBerlin, Germany; ^12^Department of Microbiology, Medical School, Aristotle University of ThessalonikiThessaloniki, Greece; ^13^National Center of Reference for Anaplasma, Babesia, Rickettsia and Theileria, Intituto Zooprofilattico Sperimentale della SiciliaSicily, Italy

**Keywords:** tick, *Anaplasma*, *flavivirus*, *Babesia*, *Borrelia*, microbiome, immunology, vaccine

## Abstract

Ticks and the pathogens they transmit constitute a growing burden for human and animal health worldwide. Vector competence is a component of vectorial capacity and depends on genetic determinants affecting the ability of a vector to transmit a pathogen. These determinants affect traits such as tick-host-pathogen and susceptibility to pathogen infection. Therefore, the elucidation of the mechanisms involved in tick-pathogen interactions that affect vector competence is essential for the identification of molecular drivers for tick-borne diseases. In this review, we provide a comprehensive overview of tick-pathogen molecular interactions for bacteria, viruses, and protozoa affecting human and animal health. Additionally, the impact of tick microbiome on these interactions was considered. Results show that different pathogens evolved similar strategies such as manipulation of the immune response to infect vectors and facilitate multiplication and transmission. Furthermore, some of these strategies may be used by pathogens to infect both tick and mammalian hosts. Identification of interactions that promote tick survival, spread, and pathogen transmission provides the opportunity to disrupt these interactions and lead to a reduction in tick burden and the prevalence of tick-borne diseases. Targeting some of the similar mechanisms used by the pathogens for infection and transmission by ticks may assist in development of preventative strategies against multiple tick-borne diseases.

## Introduction

Ectoparasites that derive nutrition through blood feeding (haematophagy) are efficient vectors of disease. Ticks are haematophagous ectoparasites of vertebrates. Approximately 10% of the 900 currently known tick species are of significant medical or veterinary importance. Besides causing direct damage associated with blood feeding and in some cases through the excretion of toxins within their saliva, the main relevance of ticks lies in the wide variety of pathogens they can transmit, including bacteria, viruses, protozoa, and helminths (Jongejan and Uilenberg, 2004). The continuous exploitation of environmental resources and the increase in human outdoor activities, which have allowed for the contact with tick vectors normally present in the field, has promoted the emergence and resurgence of tick-borne pathogens (Jongejan and Uilenberg, 2004).

As previously discussed (Beerntsen et al., 2000), the terms “vectorial capacity” and “vector competence” are often used to describe the ability of an arthropod to serve as a disease vector. However, while vectorial capacity is influenced by behavioral and environmental determinants affecting variables such as vector density, longevity, and competence, vector competence is a component of vectorial capacity that depends on genetic factors affecting the ability of a vector to transmit a pathogen (Beerntsen et al., 2000, Box 1). These genetic determinants affect traits such as tick host preferences, duration of tick attachment, tick-host-pathogen and microbiome-pathogen interactions, and susceptibility to pathogen infection (Ramamoorthi et al., 2005; Hajdušek et al., 2013; Narasimhan et al., 2014; Nuttall, 2014; Rynkiewicz et al., 2015; Vayssier-Taussat et al., 2015). Therefore, the elucidation of the mechanisms involved in tick-pathogen interactions that affect vector competence is essential for the identification of molecular drivers for tick-borne diseases, and exposes paradigms for controlling and preventing these diseases.

Box 1 **Important determinants influencing the acquisition, maintenance and transmission of pathogens by ticks.**

**Table:** 

Host range	Ticks with a wide host range such as *I. ricinus*, are naturally exposed to a greater variety of pathogens compared to ticks with a narrow host range such as *R. microplus* (Estrada-Peña et al., 2015).
Number of hosts	The potential transmission of pathogens could be limited when considering the host contact rate of 1- and 2- host ticks vs. 3-host ticks. This effect may however be partially annulled by the phenomenon of transovarial passage, when pathogens are passaged from the female to her eggs and offspring, which can subsequently infect new hosts. Argasid ticks of which the nymphs and adults take several blood meals, have a high host contact rate and could theoretically acquire or transmit pathogens from and to multiple hosts.
Midgut infection and escape barrier	The pathogen needs to pass through the midgut to reach the salivary glands and be transmitted with tick saliva, and for migration of some pathogens to the ovaries to allow transovarial pathogen passage. Mechanisms to pass the midgut infection barrier may depend on the presence and structure of specific surface receptors, such as TROSPA, to which OspA from *B. burgdorferi* adheres, allowing the spirochete to colonize the midgut (Pal et al., 2004).
Innate immune response	Pathogens need to overcome tick defense mechanisms, such as the phagocytosis of microbes by hemocytes, antimicrobial peptides and RNA interference, in order to be transmitted with tick saliva (Hajdušek et al., 2013).
Salivary gland infection and escape barrier	Pathogens must cross into the salivary glands for transmission with saliva during feeding, but little is known about the molecular mechanisms behind this entry. Once inside the salivary glands, the pathogen has to be released into the saliva stream to be transmitted. For example, *B. burgdorferi* uses tick salivary gland proteins to facilitate infection of the mammalian host (Ramamoorthi et al., 2005).
Pathogen strains	Differences between pathogen strains to infect and be transmitted by ticks have been widely reported (e.g., Kleiboeker et al., 1999; de la Fuente et al., 2001).
Tick microbiome-pathogen interactions	Microbiome play an essential role in various aspects of the arthropods life cycle and there is an increasing interest to elucidate arthropod-microbiome interactions. Perturbation of the microbiome caused changes in the integrity of the peritrophic membrane and may affect pathogen infection (Narasimhan et al., 2014).
Cross-Immunity interference	Competition between microorganisms within the tick may affect vector competence. Ticks infected with one *Rickettsia* species were for instance refractory to transovarial passage of a second *Rickettsia* species (Macaluso et al., 2002).
Abiotic factors	Abiotic factors such as temperature and relative humidity not only have a direct effect on tick development, questing activity and longevity, but temperature may also modulate pathogen development and survival in ticks (Shih et al., 1995; Estrada-Peña et al., 2011).

Although our understanding of tick-pathogen interactions is still limited, advances in this field are facilitated by the increasing number of available genomic resources, including metabolomics, transcriptomics, and proteomics datasets of various ticks and tick-borne pathogens (TBPs) (Nene et al., 2004; Ayllón et al., 2015a; Cramaro et al., 2015; Kotsyfakis et al., 2015; Villar et al., 2015a; Gulia-Nuss et al., 2016; de Castro et al., 2016), and the recently published genome from *Ixodes scapularis*, a vector of *Borrelia burgdorferi* and *Anaplasma phagocytophilum* in North America (Gulia-Nuss et al., 2016). Together with tools such as tick cell lines and the widespread adaptation of RNA interference (RNAi) to study tick gene function (Bell-Sakyi et al., 2007; de la Fuente et al., 2007), this has opened exciting possibilities to identify determinants affecting tick vector competence.

**Figure 1:**
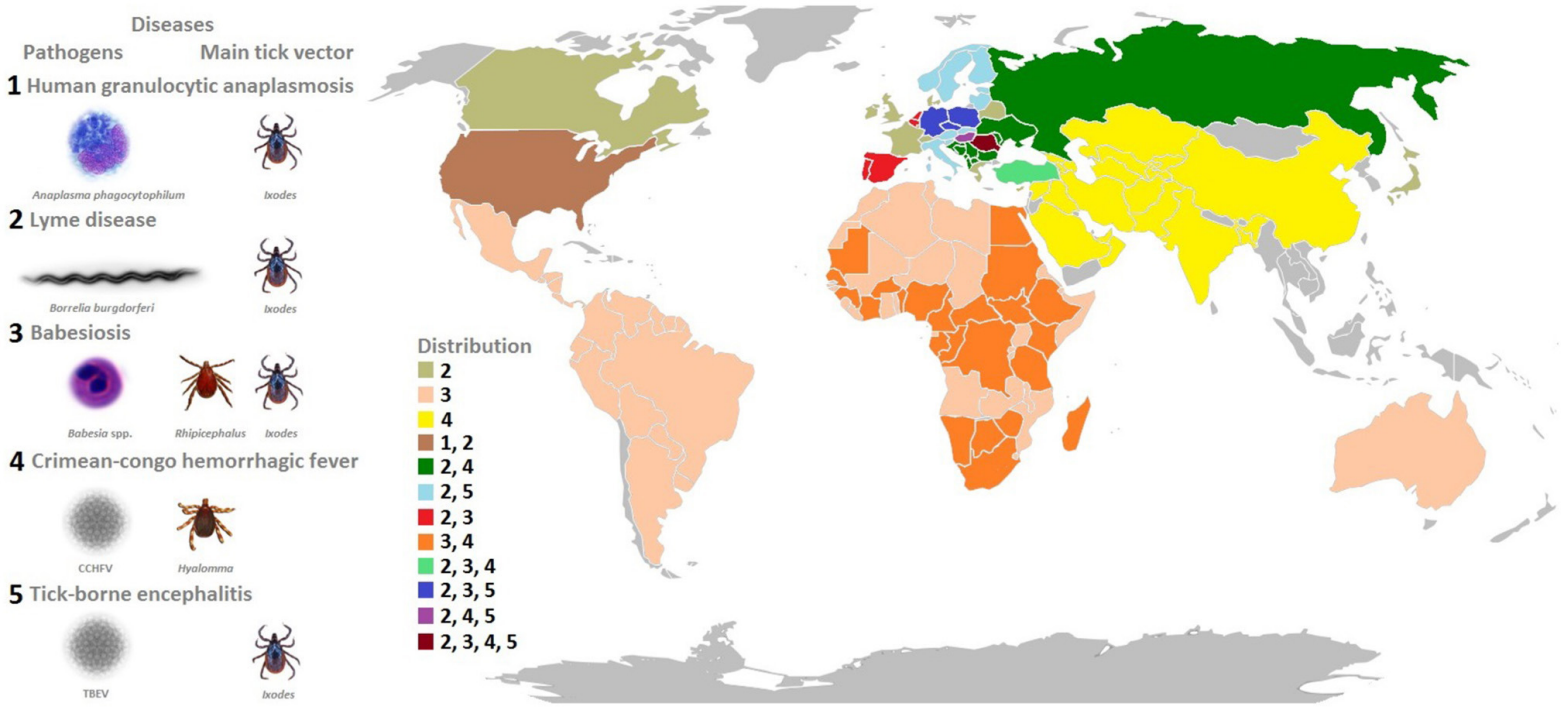
**Model organisms: tick-borne pathogens that constitute a growing burden for human and animal health**. The pathogens covered in this review include bacteria (*A. phagocytophilum* and *B. burgdorferi*), viruses (Crimean-Congo hemorrhagic fever virus, tick-borne encephalitis virus), and protozoa (*Babesia* spp.) transmitted by hard ticks (Ixodidae). The most prevalent diseases caused by these pathogens, main tick vectors, and disease distribution worldwide is shown in the figure.

Most studies of tick-pathogen interactions focus on certain pathogens (e.g., de la Fuente et al., 2016) or on certain aspects of these interactions (e.g., Hajdušek et al., 2013). However, for a better understanding of tick-pathogen molecular interactions and their role in vector competence, a comprehensive analysis involving major pathogens is crucial. In this review, we provide an overview of tick-pathogen molecular interactions for TBPs that constitute a growing burden for human and animal health (Figure 1). Additionally, the impact of tick microbiome on these interactions was considered to further contribute to the identification of molecular drivers affecting vector competence and the development of novel control and prevention strategies for tick-borne diseases.

## Model microorganisms

In this review, we used different tick-borne microorganisms including bacteria (*A. phagocytophilum* and *B. burgdorferi*), viruses (Crimean-Congo hemorrhagic fever virus, tick-borne encephalitis virus, and louping ill virus), and protozoa (*Babesia* spp.) to illustrate their impact on vector competence, behavior and transmission (Figure 1).

### Bacteria

*Anaplasma phagocytophilum* is an obligate intracellular rickettsial pathogen vectored primarily by *Ixodes* spp. and causes human granulocytic anaplasmosis (HGA), equine, and canine granulocytic anaplasmosis, and tick-borne fever (TBF) (de la Fuente et al., 2008). In the vertebrate host, *A. phagocytophilum* infects neutrophils where the pathogen multiplies within a parasitophorous vacuole or morula (Ayllón et al., 2015a; Severo et al., 2015). In the absence of transovarial passage, ticks must acquire infection in each generation during a bloodmeal. *A. phagocytophilum* initially infects tick midgut cells and then subsequently develops in the salivary glands for transmission to susceptible hosts during tick feeding. Bacteria from the *B. burgdorferi* sensu lato complex are transmitted by Ixodid ticks and cause various symptoms associated with Lyme disease (Radolf et al., 2012). *B. burgdorferi* s.l. are acquired by larvae or nymphs from an infected host as they are not transovarially transmitted (Rollend et al., 2013). In the tick, spirochetes colonize the midgut and then traverse into the hemocoel and migrate to salivary glands for transmission during tick feeding (Pal et al., 2004; Ramamoorthi et al., 2005; Zhang L. et al., 2011; Coumou et al., 2016).

### Viruses

Ticks transmit a range of viruses that are of significant public and veterinary health concern (Table 1). It is estimated that these viruses spend over 95% of their life cycle within the tick vector. Tick-borne encephalitis virus (TBEV) causes neurological disease in humans, whereas louping ill virus (LIV) causes neurological disease in sheep (Labuda and Nuttall, 2003). Ixodid ticks transmit these viruses to particular host species through a bite (Doherty and Reid, 1971; Mansfield et al., 2016). Crimean-Congo hemorrhagic fever virus (CCHFV) is transmitted to humans by the bite of infected ticks (*Hyalomma* spp. are the most competent vectors) or by direct contact with blood or tissues of viremic patients or animals, causing a disease characterized by fever, headache, myalgia, and hemorrhagic manifestations (Papa, 2010). If the appropriate receptors are present in the tick, following a blood meal TBEV and CCHFV enter vector host cells by endocytosis (Labuda and Nuttall, 2003; Simon et al., 2009; Garrison et al., 2013; Shtanko et al., 2014; Suda et al., 2016). These viruses replicate in the lining of the tick midgut where they disseminate to the hemolymph and subsequently infect different tissues reaching the highest titers in the salivary glands and reproductive organs to exit the cell via exocytosis (Dickson and Turell, 1992).

**Table 1:** **Viruses transmitted by ticks of medical or veterinary importance**.

**Virus (abbreviation)**	**Family/Genus**	**Principal vector**	**Species affected**	**Endemic presence**
Alkhurma hemorrhagic fever virus (AHFV)	*Flaviviridae/Flavivirus*	*Ornithidoros savigny*	Humans	Saudi Arabia
African swine fever virus (ASFV)	*Asfarviridae/Asfivirus*	*Ornithodoros moubata*	Pigs	Africa
Colorado tick fever virus (CTFV)	*Reoviridae/Coltivirus*	*Dermacentor andersoni*	Humans	North America
Crimean-Congo haemorrhagic fever virus (CCHFV)	*Bunyaviridae/Nairovirus*	*Hyalomma* spp.	Humans	Africa/Asia/Southern Europe
Kyasanur Forrest virus (KFV)	*Flaviviridae/Flavivirus*	*Haemaphysalis spingera*	Humans	India
Louping ill virus (LIV)	*Flaviviridae/Flavivirus*	*Ixodes ricinus*	Sheep/Grouse	British Isles
Nairobi sheep disease virus (NSDV)	*Bunyaviridae/Nairovirus*	*Rhipicephalus appendiculatus*	Sheep	Africa
Omsk Hemorrhagic fever virus (OHFV)	*Flaviviridae/Flavivirus*	*Dermacentor reticulatus*	Humans	Asia
Powassan virus (POWV)	*Flaviviridae/Flavivirus*	*Ixodes cookei*	Humans	North America/Russia
Tick-borne encephalitis virus (TBEV)	*Flaviviridae/Flavivirus*	*I. ricinus/Ixodes persulcatus*	Humans	Europe/Asia

*Table adapted from Labuda and Nuttall (2003) and Johnson et al. (2012)*.

### Protozoa

*Babesia* spp. are tick-borne Apicomplexan protozoans which invade vertebrate host erythrocytes, where all hemoparasite phases occur (Yokoyama et al., 2006; Chauvin et al., 2009; Florin-Christensen and Schnittger, 2009). *Babesia bovis* and *Babesia bigemina*, transmitted mainly by *Rhipicephalus microplus* and *Rhipicephalus annulatus*, are considered the most important species for their great economic impact on the cattle industry. Humans are accidental hosts, but human babesiosis caused by *Babesia microti* is now considered an emerging zoonosis as cases are increasing yearly (Schnittger et al., 2012). Ticks become infected with *Babesia* parasites when ingesting blood cells containing piroplasms, which develop into male and female gametes in the tick midgut (Uilenberg, 2006). The zygotes then multiply and invade numerous tick organs including the ovaries, which results in transovarial passage for some species such as *B. bovis* and *B. bigemina* but not *B. microti* (Uilenberg, 2006). When ticks attach to a new host, the sporozoites mature and the parasites are transmitted with tick saliva and infect red blood cells (Uilenberg, 2006).

## Biological processes involved in tick-pathogen interactions

The objective of this paper is to review the information available on tick-pathogen molecular interactions and their role in vector competence. To address this objective, we discussed the main biological processes involved in tick-pathogen interactions. Additionally, the impact of tick microbiome on these interactions was considered. Although host-tick and host-pathogen molecular interactions also affect vector competence, this review focuses on tick-pathogen interactions for the identification of molecular drivers affecting vector competence that may result in the identification of tick-derived and pathogen-derived antigens for the development of novel control and prevention strategies for tick-borne diseases.

### Role of bacterial proteins in tick-pathogen interactions

Tick-pathogen protein-protein interactions play a crucial role during pathogen infection, persistence and transmission. The analysis of *A. phagocytophilum* proteins differentially represented during infection in ticks demonstrated that heat shock protein 70 (HSP70) and major surface protein 4 (MSP4) interact and bind to tick cells, thus playing a role in tick-pathogen interactions (Villar et al., 2015b). The type IV secretion system (T4SS) was proposed to be involved in the secretion of HSP70 and the MSP4 interaction with tick cells may induce the secretion of vesicles at the phagocytic cup to aid in adhesin secretion for rickettsial infection of tick cells (Villar et al., 2015b). Recent results have advanced our understanding of the molecular factors that are involved in the acquisition, persistence and transmission of *B. burgdorferi* in ticks (Rosa et al., 2005; Kung et al., 2013). An important protein involved in spirochete colonization of the tick midgut is the outer surface protein A (OspA), which binds to the tick receptor for OspA (TROSPA) (Pal et al., 2004). An *I. scapularis* dystroglycan like protein (ISDLP) as well as a tick receptor for the *B. burgdorferi* protein BBE31 (TRE31) help spirochetes traverse from the tick midgut into the hemocoel (Zhang L. et al., 2011; Coumou et al., 2016). *B. burgdorferi* outer surface protein C (OspC), produced when bacteria leave the tick midgut, binds to tick salivary protein 15 (Salp15) (Ramamoorthi et al., 2005), providing protection against mammalian antibody/complement-mediated immune response during bacterial transmission (Garg et al., 2006; Schuijt et al., 2011a). The TROSPA homolog in the *B. bigemina* vectors, *R. microplus*, and *R. annulatus* was proposed to be a putative receptor for *Babesia* ligands based on the decrease in infection after RNAi and vaccination experiments targeting this protein (Antunes et al., 2012; Merino et al., 2013). Flaviviruses and CCHFV enter vertebrate and vector host cells by attachment of viral envelope proteins to host receptors, which activates the actin-dependent clathrin-mediated endocytic pathway (Labuda and Nuttall, 2003; Simon et al., 2009; Garrison et al., 2013).

### Tick cytoskeleton

Intracellular bacteria induce cytoskeletal rearrangement to establish infection (Ireton, 2013). In *I. scapularis, A. phagocytophilum* remodels tick cytoskeleton by altering the ratio between monomeric globular G actin and filamentous F actin to facilitate infection through selective regulation of gene transcription in association with the RNA polymerase II and the TATA-binding protein (Sultana et al., 2010). In *I. scapularis* midgut cells, the up-regulation of Spectrin alpha chain or Alpha-fodrin in response to infection results in cytoskeleton remodeling that is used by *A. phagocytophilum* to facilitate infection (Ayllón et al., 2013, Figure 2A). Although not functionally characterized, a proteomics analysis in *I. ricinus* tick salivary glands showed the under-representation of cytoskeleton proteins in response to *Borrelia* infection, suggesting that some *Borrelia* strains promote a cytoskeleton rearrangement in ticks (Cotté et al., 2014, Figure 2B).

**Figure 2:**
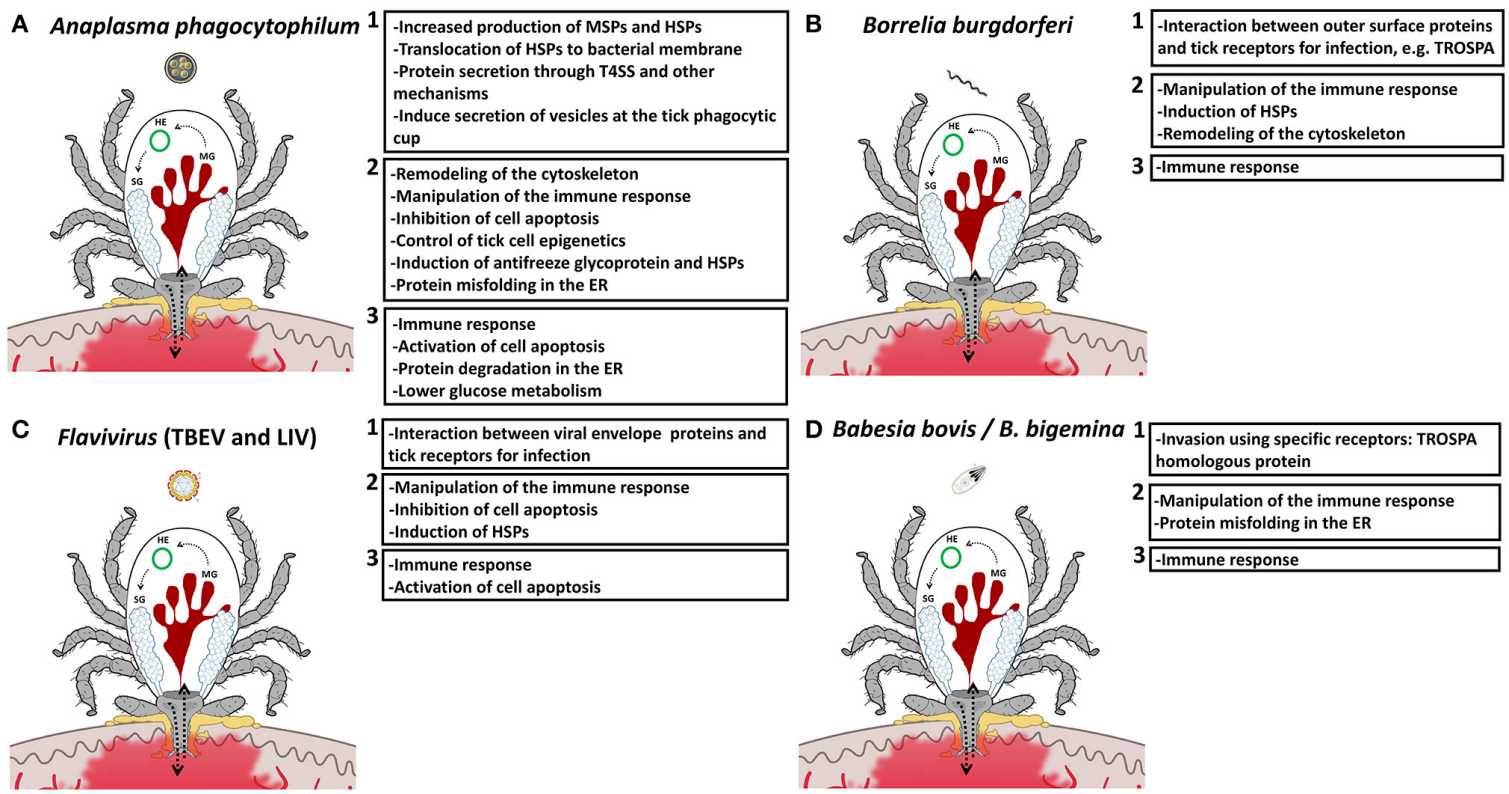
**Tick-pathogen molecular interactions. (A)** *A. phagocytophilum* **(B)** *B. burgdorferi* s.l., **(C)** TBEV, and **(D)** *B. bovis/B. bigemina* activate mechanisms (panel 1) and manipulate tick protective responses and other biological processes in order to facilitate infection (panel 2), while ticks respond to limit pathogen infection and preserve feeding fitness and vector competence for survival of both ticks and pathogens (panel 3). MG, midgut; HE, hemocyte; SG, salivary gland; MSPs, major surface proteins; HSPs, heat shock proteins; ER, endoplasmic reticulum.

### Tick cell apoptosis

Apoptosis is an intrinsic immune defense mechanism in response to microbial infection that results in reduction of infected cells, but several pathogens have developed different strategies to inhibit cell apoptosis in order to enhance their infection, replication and survival (Ashida et al., 2011). Infection of tick salivary glands with *A. phagocytophilum* results in inhibition of the intrinsic apoptosis pathway through porin down-regulation, favoring bacterial infection (Ayllón et al., 2015a). Tick cells respond to infection via activation of the extrinsic apoptosis pathway, which limits *A. phagocytophilum* infection and promotes tick survival (Ayllón et al., 2015a). In tick midguts, *A. phagocytophilum* infection results in activation of the Janus kinase/signal transducers and activators of transcription (JAK/STAT) pathway, which inhibits apoptosis and promotes pathogen infection (Ayllón et al., 2015a). The ISE6 cultured cells, derived from embryonic *I. scapularis*, have provided a model for tick hemocyte responses to pathogen infection. In this cell line, *A. phagocytophilum* infection promotes protein misfolding in the endoplasmic reticulum (ER), counteracting the tick cell response to infection. However, tick cells respond by activating protein targeting and degradation, which reduces ER stress and apoptosis, thus favoring *A. phagocytophilum* infection (Villar et al., 2015a). Additionally, *A. phagocytophilum* may benefit from the tick cells ability to limit pathogen infection through phosphoenolpyruvate carboxykinase (PEPCK) inhibition that results in lower glucose metabolism and the reduction in the availability of essential metabolites for bacterial growth, which leads to the inhibition of cell apoptosis that increases infection in tick cells (Villar et al., 2015a). These results show that the inhibition of tick cell apoptosis is a physiologically relevant mechanism used by *A. phagocytophilum* to facilitate infection and multiplication in both tick and vertebrate host cells (de la Fuente et al., 2016, **Figure 4**). Infection of *I. ricinus* cells with flaviviruses leads to the differential expression of a large number of genes involved in a variety of cellular functions, including up-regulation of genes such as *cytochrome c* associated with cellular stress and apoptosis (Mansfield et al., 2017). However, the lack of detection of *caspase* genes, and the up-regulation of genes that inhibit apoptosis (including *hsp70*) suggest that flavivirus infection inhibits tick cell apoptosis in order to promote cell survival during infection as previously shown for *A. phagocytophilum* (Ayllón et al., 2015a; Alberdi et al., 2016).

### Tick innate immune response

Tick vector competence is influenced by the ability of transmitted pathogens to evade tick innate immune response (Hajdušek et al., 2013). Several humoral and cell-mediated immune response pathways are involved in tick innate immunity, and play a role in defense to *Anaplasma, Borrelia*, flavivirus, and *Babesia* infection or are manipulated by pathogens to facilitate infection (Turell, 2007; Hajdušek et al., 2013; Mansfield et al., 2017, Figure 2). With respect to the tick innate immune response, *A. phagocytophilum* subverts tick RNAi by mechanisms other than reduction of Tudor staphylococcal nuclease (Tudor-SN) levels to preserve a protein that is important for tick feeding (Ayllón et al., 2015b). In contrast, Subolesin (SUB), also involved in tick innate immune response for limiting pathogen infection (Naranjo et al., 2013; de la Fuente and Contreras, 2015), is not manipulated by *A. phagocytophilum*. SUB has been shown to be required for tick feeding and reproduction and for pathogen infection, and therefore the preservation of this protein is important for both tick and pathogen survival (de la Fuente and Contreras, 2015). In *I. scapularis*, the x-linked inhibitor of apoptosis protein (XIAP) interacts with the E2 conjugating enzyme Bendless affecting positive and negative regulators of the immune deficiency (IMD) pathway resulting in protection against infection by *A. phagocytophilum* (Severo et al., 2013).

After molting, tick nymphs attach and start feeding, displaying an altered midgut transcriptome when infected with *B. burgdorferi* (Rudenko et al., 2005). Some of the genes affected by infection include innate immune factors (defensin and thioredoxin peroxidase) that possibly limit tick *Borrelia* infection. Tick salivary protein 20 (Salp20) belongs to a protein family with complement-inhibitory activity that blocks the host alternative complement pathway and assists in *Borrelia* transmission (Hourcade et al., 2016). Tick salivary lectin pathway inhibitor (TSLPI) inhibits the human lectin complement pathway by interfering with the mannose binding lectin activity and enables transmission of *Borrelia* by protecting it from complement-mediated killing (Schuijt et al., 2011b; Wagemakers et al., 2016). Recently, Smith et al. (2016) showed that *I. scapularis* respond to interferon gamma acquired in the blood meal when parasitizing on *B. burgdorferi*-infected mice, leading to the up-regulation of the Rho-like GTPase and induction of antimicrobial peptides to inhibit pathogen infection.

Preliminary studies focusing on transcriptomic changes induced by TBEV infection of *I. scapularis* and *I. ricinus* cells have revealed the role of particular proteins within tick innate immune pathways that act to control infection (Weisheit et al., 2015). A similar approach has identified this response in tick cells infected with LIV and TBEV, with a range of transcripts being up and down-regulated (Weisheit et al., 2015; Mansfield et al., 2017). Flavivirus infection also induced transcripts associated with activation of innate immune pathways in tick cells, including JAK/STAT and Mitogen-activated protein kinase (MAPK) pathways (Mansfield et al., 2017), with additional up-regulation of genes with host resistance functions, including FK506 binding protein (FKBP) and the antiviral helicase Slh1 (Mansfield et al., 2017, Figure 2C). CCHFV is capable of evading the tick innate immune response. Following intracoelomic CCHFV inoculation, virus titers in male and female ticks are the same and infection rates and titers in salivary glands, ovaries, and testes increase upon blood feeding (Dickson and Turell, 1992). Therefore, viral replication in tissues associated with possible CCHFV transmission in infected ticks may be stimulated by attachment and feeding on susceptible hosts. This might reduce the stress induced by viral replication while ticks are waiting to find a vertebrate host, but increase the potential for viral transmission once the host is infested (Turell, 2007).

Using different methodologies, some molecules have been identified as being implicated in tick-*Babesia* interactions (Hajdušek et al., 2013). Genes involved in immunity, stress, and defense responses showed up-regulation in response to *B. bovis* infection (Heekin et al., 2012), while genes encoding for calreticulin, kunitz-type serine protease inhibitors and microplusin which exhibits antimicrobial activity, were differentially expressed in *B. bovis/B. bigemina* infected *Rhipicephalus* ticks (Rachinsky et al., 2007; Antunes et al., 2012; Heekin et al., 2013; Lu et al., 2016). Tick SUB (Almazán et al., 2005) was shown to be up-regulated in *B. microti* inoculated intrahemocoelically into *Rhipicephalus haemaphysaloides* (Lu et al., 2016) and *B. bigemina*-infected *R. microplus* (Merino et al., 2013) (Figure 2D). The putative role of SUB in *B. bigemina* infection in ticks was supported by showing a decrease in pathogen levels in ticks fed on cattle immunized with recombinant SUB (Merino et al., 2013).

### Tick cell epigenetics

Intracellular pathogens manipulate the transcriptional programs of their host cells via epigenetic mechanisms, leading to stress, and inflammatory responses (Gómez-Díaz et al., 2012). Recently, *A. phagocytophilum* was shown to manipulate tick cell epigenetics to increase the levels of the histone modifying enzymes (HMEs), histone acetyltransferases (HATs; 300/CBP), and histone deacetylases (HDACs and Sirtuins) resulting in the inhibition of cell apoptosis to facilitate pathogen infection and multiplication (Cabezas-Cruz et al., 2016). The results of this study suggested that a compensatory mechanism might exist by which *A. phagocytophilum* differentially manipulates tick HMEs to regulate transcription and apoptosis in a tissue-specific manner to facilitate infection but preserving tick fitness to guarantee survival of both pathogens and ticks (Cabezas-Cruz et al., 2016). As previously discussed (Cabezas-Cruz et al., 2016), the mechanisms by which *A. phagocytophilum* affects tick cell epigenetics is unknown but effector proteins such as AnkA, secreted through T4SS or other secretion mechanisms probably control it (Garcia-Garcia et al., 2009a,b; Rennoll-Bankert et al., 2015). It has been previously demonstrated that *A. phagocytophilum* AnkA recruits host histone deacetylase 1 (HDAC1) and modifies neutrophils gene expression (Garcia-Garcia et al., 2009a,b; Rennoll-Bankert et al., 2015). Interestingly, the homolog of HDAC1 in *I. scapularis* was overrepresented upon *A. phagocytophilum* infection in tick salivary glands (Cabezas-Cruz et al., 2016). It remains to be tested whether *A. phagocytophilum* AnkA plays the same role in ticks as in vertebrate neutrophils.

### Effect of pathogen infection on tick fitness

The characterization of *I. scapularis*-*A. phagocytophilum* molecular interactions revealed complex responses by both ticks and pathogens that were necessary for maintenance of tick health while ensuring robust vector capacity (Ayllón et al., 2015a; Villar et al., 2015a; Gulia-Nuss et al., 2016; de la Fuente et al., 2016). Several lines of evidence suggest that tick-pathogen associations evolved to form “*intimate epigenetic relationships*” that have the potential to increase tick fitness (Cabezas-Cruz et al., 2017). At the tick-pathogen interface, *A. phagocytophilum* induces an antifreeze glycoprotein (IAFGP) and heat shock proteins (HSPs) to increase tick survival and feeding fitness (Neelakanta et al., 2010; Busby et al., 2012). Neelakanta et al. (2010) demonstrated that *I. scapularis* ticks infected with *A. phagocytophilum* show enhanced fitness against freezing injury due to the induced expression of IAFGP. They further showed that improved survival of infected ticks correlated with higher bacterial infection, therefore providing a direct link between pathogen infection and tick fitness in unfavorable ecological conditions. The fact that *Borrelia* and TBEV-infected ticks choose a higher questing height suggests that these pathogens help ticks to survive under dry conditions. In agreement with this hypothesis, *I. ricinus* infected by *B. burgdorferi* move less toward a humid environment and their survival is higher in highly desiccating conditions (Hermann and Gern, 2010; Herrmann and Gern, 2012). The tick histamine release factor (tHRF), up-regulated in *B. burgdorferi*-infected *I. scapularis* during feeding, facilitates tick engorgement and *B. burgdorferi* infection by increasing the blood flow to the tick-bite site and modulating vascular permeability (Dai et al., 2010).

## Tick-microbiome interactions

The recent development of high-throughput next generation sequencing technologies has highlighted the complexity of the tick microbiome that includes both pathogens and potential symbionts (Vayssier-Taussat et al., 2015). It is readily apparent that interactions frequently occur among tick microbial communities, as relationships between microorganisms existing in one environment can be competitive, exclusive, facilitating, or absent, with many potential implications for human and animal health that remain to be elucidated (Ahantarig et al., 2013; Vayssier-Taussat et al., 2015). Both positive and negative associations have been reported for pathogens (Mather et al., 1987; de la Fuente et al., 2003). However, the role of tick endosymbionts in pathogen transmission has only been studied in a few selected bacterial and tick species.

Symbionts may confer crucial and diverse benefits to their hosts, playing nutritional roles, or affecting fitness, development, reproduction, defense against environmental stress, and immunity (Ahantarig et al., 2013). *Coxiella*-like endosymbionts are believed to be the most common vertically transmitted agents in hard ticks (Bernasconi et al., 2002; Lee et al., 2004; Clay et al., 2008; Bonnet et al., 2013; Cooper et al., 2013). In *Amblyomma americanum*, the removal of *Coxiella* symbionts following antibiotic treatment reduced tick offspring production and increased time to oviposition (Zhong et al., 2007). In *I. ricinus* (Lo et al., 2006; Sassera et al., 2006; Montagna et al., 2013), *Candidatus* Midichloria mitochondrii is an intra-mitochondrial bacterium that has also been detected in other tick genera (Harrus et al., 2011; Williams-Newkirk et al., 2012). It has been ascribed a possible helper role in tick molting processes (Zchori-Fein and Bourtzis, 2011, Figure 3). *Rickettsia*-like symbionts have also been reported to infect hard ticks from several genera (Baldridge et al., 2004; Clay et al., 2008; Liu et al., 2013). One study reported that *Rickettsia*-infected *Dermacentor variabilis* have slightly greater motility than uninfected ticks, indirectly influencing disease risk (Kagemann and Clay, 2013). *Francisella*-like symbionts have been reported in several hard tick genera (Venzal et al., 2008; Ivanov et al., 2011; Michelet et al., 2013), but their effect on tick fitness and biology remains unknown. Being able to manipulate host reproduction and then to affect vector populations, *Wolbachia* spp. have also been identified in several hard tick genera (Engelstadter and Hurst, 2007; Andreotti et al., 2011; Reis et al., 2011; Zhang X. et al., 2011). Their role in pathogen transmission requires further attention, as reports suggest that this bacterium can protect some arthropods against microbial infections (Martinez et al., 2014). In *I. ricinus, Wolbachia pipientis* is known to be associated with the hymenoptera tick endoparasitoid *Ixodiphagus hookeri* (Plantard et al., 2012; Bohacsova et al., 2016), and *Arsenophonus* spp. symbionts (Dergousoff and Chilton, 2010). The latter, detected in several tick species (Clay et al., 2008; Dergousoff and Chilton, 2010; Reis et al., 2011), are responsible for sex-ratio distortion in arthropods, and some studies suggest that they can affect host-seeking success by decreasing tick motility in *A. americanum* and *D. variabilis* (Kagemann and Clay, 2013). Lastly, some *Spiroplasma* spp. detected in *Ixodes* spp. such as *Spiroplasma ixodetis* (Tully et al., 1995) may cause sex-ratio distortion in some insect species via male killing (Tabata et al., 2011).

**Figure 3:**
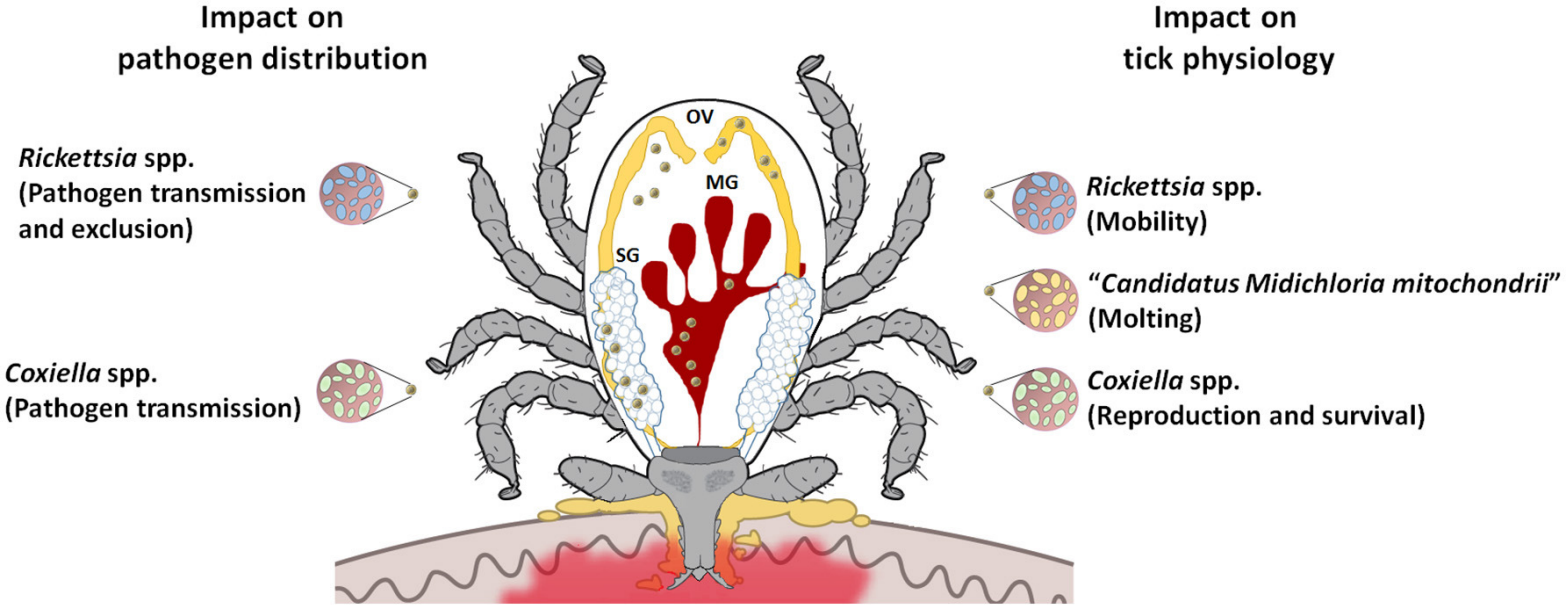
**Possible impact of tick microbiome on pathogen transmission**. Tick microbiome may affect pathogen transmission either directly via nutrient competition or induced/reduced immunity, or indirectly by affecting tick populations (viability, reproduction) or fitness (affecting host-seeking success). MG, midgut; SG, salivary gland; OV, ovaries.

Recently, Abraham et al. (2017) showed how *A. phagocytophilum* manipulates *I. scapularis* tick microbiota to promote infection. Firstly, they showed that IAFGP, apart from protecting ticks against cold injury (see above), has antimicrobial activity against biofilm-forming bacteria, particularly *Staphylococcus aureus* and *Enterococcus faecalis*. They further showed that by targeting biofilm-forming bacteria, *A. phagocytophilum* modifies the composition of gut microbiota and alters tick midguts permeability, which results in higher *A. phagocytophilum* infection in the vector (Abraham et al., 2017). Regarding the relationship between symbionts and pathogens, exclusion has been reported in Rickettsiales, which may be due to intra-family bacterial cross-immunity. Exclusion has been documented in *Dermacentor* ticks infected with *Rickettsia peacockii* or *Rickettsia montana* that limits *Rickettsia rickettsii* and *Rickettsia rhipicephali* distribution, respectively (Burgdorfer et al., 1981; Macaluso et al., 2002, Figure 3). It has also been reported that *I. scapularis* male ticks infected by a rickettsial endosymbiont had significantly lower rates of infection by *B. burgdorferi* than symbiont-free males, thus evidencing interactions among microbial species (Steiner et al., 2008). Further research showed that perturbation of the midgut microbiome in *I. scapularis* influences *B. burgdorferi* colonization of ticks through a transcriptional mechanism resulting in lower expression of peritrophin, which perturbs the integrity of the peritrophic matrix (Narasimhan et al., 2014). In *A. americanum*, the presence of *Coxiella*-related symbionts seems to influence *Ehrlichia chaffeensis* transmission (Klyachko et al., 2007), and infection with *Arsenophonus* appears to be negatively correlated with the frequency of *Rickettsia* sp. infection (Clay et al., 2008, Figure 3).

## Conclusions and future directions for the control of tick-borne diseases

Over millions of years, arthropod vectors have co-evolved with a variety of microorganisms including bacteria, viruses, and protozoa to the point where they appear to co-exist with little impact on the vector (Beerntsen et al., 2000; Estrada-Peña et al., 2015; de la Fuente et al., 2015). These arthropods have become efficient vectors of pathogens to humans and other vertebrate hosts that are susceptible to infection and disease.

Present results show that different pathogens have developed similar strategies such as manipulation of the immune response to infect ticks and facilitate multiplication and transmission. Some of these strategies may be used by pathogens to infect both ticks and mammalian hosts (de la Fuente et al., 2016). Additionally, recent evidence demonstrates that the microbiome has an effect on tick fitness and pathogen infection and transmission, highlighting the importance of tick-microbiome interactions for vector competence. Overall, these results illustrate how pathogens activate mechanisms and manipulate tick protective responses and other biological processes in order to facilitate infection, while ticks respond to limit pathogen infection and preserve feeding fitness and vector competence for survival of both ticks and pathogens. However, how different molecular mechanisms make certain tick species suitable vectors for certain pathogens is still not fully characterized. The presence of tick receptors that are pathogen-specific affects vector competence for these pathogens, but other mechanisms are probably also involved in this process. Furthermore, the biological processes involved in tick-pathogen interactions are also affected in other arthropod vectors (Box 2).

Box 2 **Are the biological processes involved in tick-pathogen interactions unique for ticks?**

The answer to this question is that several of the processes involved in tick-pathogen interactions have also been identified in other vector-pathogen interactions (see for example, Beerntsen et al., 2000; Vlachou et al., 2005; Wang et al., 2010; Gómez-Díaz et al., 2012; Sabin et al., 2013; Ramphul et al., 2015; Eng et al., 2016; Shaw et al., 2017). For example, as described in ticks, receptor-ligand-like interactions mediate pathogen recognition and infection in mosquitoes (Beerntsen et al., 2000). Remodeling of the cytoskeleton seems to be a general mechanism for tick pathogen infection (Cotté et al., 2014; de la Fuente et al., 2016). Pathogens such as Dengue virus (DENV), West Nile virus (WNV), and *Plasmodium* parasites also affect mosquito cytoskeleton during infection (Vlachou et al., 2005; Wang et al., 2010). The finding that some pathogens manipulate tick immune response to facilitate infection has been also reported in mosquitoes infected with *Plasmodium falciparum* (Beerntsen et al., 2000). Similarly, the expression of immune response genes such as those involved in the JAK/STAT pathway may serve to limit bacterial and fungal proliferation in fruit fly and mosquitoes (Beerntsen et al., 2000). Apoptosis plays an important role in tick-pathogen interactions (de la Fuente et al., 2016). While inhibition of cell apoptosis by pathogens facilitates infection, host cell response may activate alternative apoptotic pathways to limit infection (de la Fuente et al., 2016). These findings have been also described in for example *Aedes aegypti* and *Anopheles gambiae* mosquitoes infected with DENV and *P. falciparum*, respectively (Ramphul et al., 2015; Eng et al., 2016). The control of tick cell epigenetics by *A. phagocytophilum* has been proposed as a mechanism used by the pathogen to facilitate infection and multiplication (Cabezas-Cruz et al., 2016). Similar mechanisms have been described to operate at the mosquito-*Plasmodium* interface (Gómez-Díaz et al., 2012).

**Figure 4:**
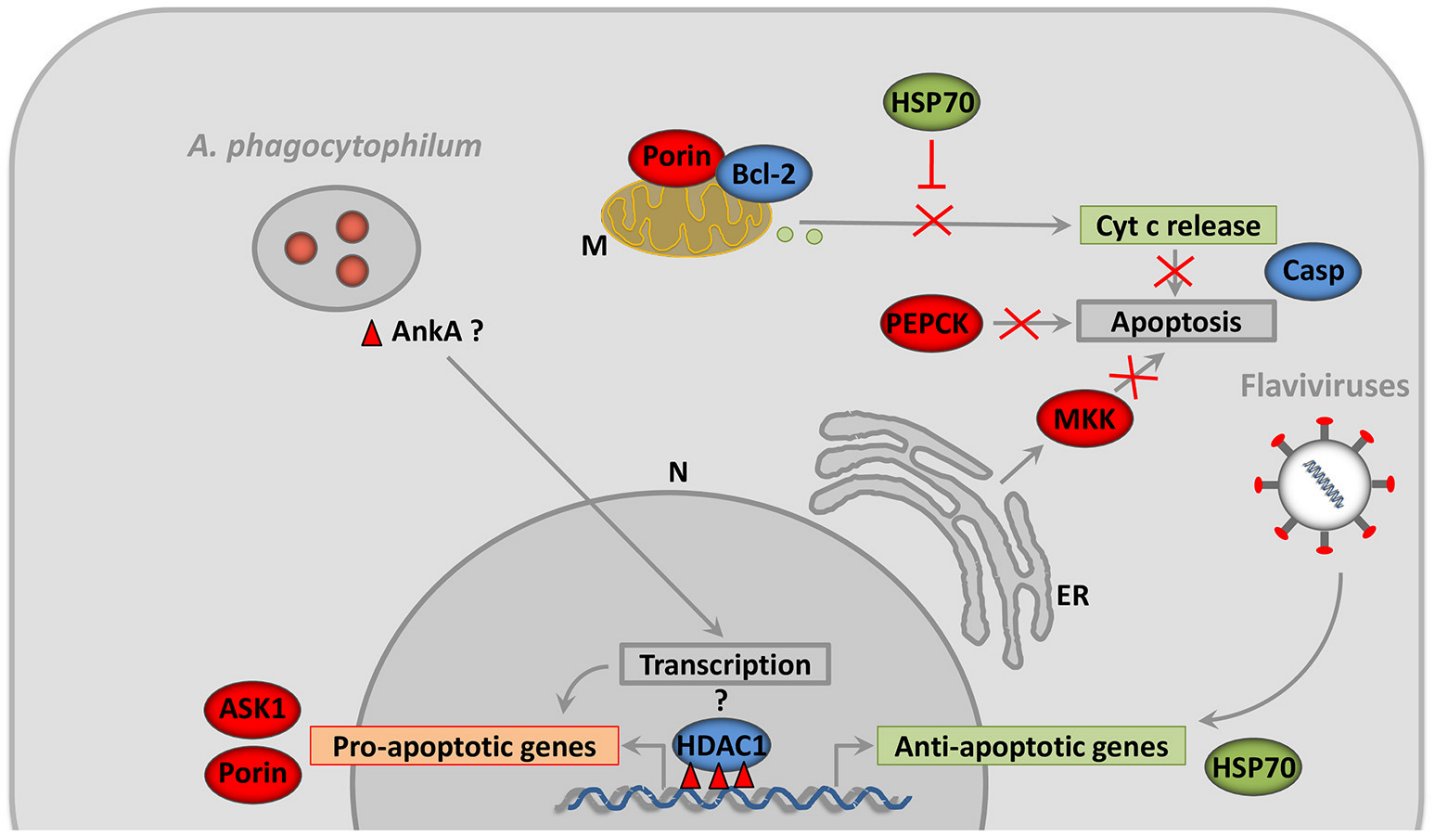
**Pathogens inhibit vector cell apoptosis by different mechanisms**. After infection of tick salivary glands, *A. phagocytophilum* inhibit apoptosis by decreasing the expression of the pro-apoptotic genes coding for proteins such as ASK1 and Porin. Porin down-regulation is associated with the inhibition of mitochondrial Cyt c release (Ayllón et al., 2015a). In contrast, *A. phagocytophilum* infection does not affect Bcl-2 levels, probably because this protein but not Porin is essential for tick feeding (Ayllón et al., 2015a). *A. phagocytophilum* also induces ER stress in tick cells which play a role in reducing the levels of MKK that inhibits apoptosis (Villar et al., 2015a). Another interesting mechanism of *A. phagocytophilum* to inhibit apoptosis is the manipulation of glucose metabolism by reducing the levels of PEPCK (Villar et al., 2015a). The capacity of *A. phagocytophilum* to downregulate gene expression in neutrophils was associated with HDAC1 recruitment to the promoters of target genes by the ankyrin repeat protein AnkA (Garcia-Garcia et al., 2009a,b; Rennoll-Bankert et al., 2015). Tick HDAC1 is overrepresented in *A. phagocytophilum*-infected salivary glands and chemical inhibition of this protein decreases *A. phagocytophilum* burden in tick cells (Cabezas-Cruz et al., 2016). Infection of tick cells with flaviviruses results in the up-regulation of genes such as *hsp70* that inhibit apoptosis (Mansfield et al., 2017). N, Nucleus; M, Mitochondria; ER, Endoplasmic Reticulum; Cyt c, Cytochrome c; ASK1, Apoptosis signal-regulating kinase 1; MKK, Mitogen-activated Protein Kinase; HDAC1, Histone Deacetylase 1; AnkA, Ankyrin A; PEPCK, Phosphoenolpyruvate Carboxykinase; FOXO, Forkhead box O; Hid, Head involution defective; JNK, Jun amino-terminal kinases; Casp, caspases. The molecules and processes represented in green are up-regulated, while those represented in red are down-regulated in response to infection. The activity of the molecules represented in blue varies in response to infection.

However, the functional mechanisms by which these processes are affected at the vector-pathogen interface may vary between pathogen and vector species (Figure 4). The limited information available on the functional characterization of these processes in ticks and other arthropods limits the scope of the comparative analysis between different vectors. Nevertheless, recent results support that in some cases the protein function described in model insect species may be different in the evolutionarily distant ticks. Differences in vector competence may be genetically encoded by differences in the immune response pathways operating at each vector-pathogen interaction (Baxter et al., 2017). For example, Tudor-SN, a conserved component of the basic RNAi machinery with a variety of functions including immune response and gene regulation, is involved in defense against infection in *Drosophila* (Sabin et al., 2013) but not in ticks (Ayllón et al., 2015b). The IMD pathway is involved in protection against infection in arthropods, but recent results support the existence of two functionally distinct IMD circuits in insects and ticks (Shaw et al., 2017). Future comparative analyses between different vector species will provide additional information on the functional implication of the different biological processes in vector-pathogen interactions and vector competence (Gerold et al., 2017).

The identification of the molecular drivers that promote tick survival, spread, and pathogen transmission provides the opportunity to disrupt these processes and lead to a reduction in tick burden and prevalence of tick-borne diseases. Targeting some of the similar mechanisms used by the pathogens for infection and transmission by ticks may be used to develop strategies against multiple tick-borne diseases. As shown for *B. burgdorferi* OspA (Gomes-Solecki, 2014), pathogen-derived proteins involved in interactions with tick cells and playing a role during infection provide targets for development of novel control strategies for pathogen infection and transmission. Similarly, tick-derived antigens such as SUB involved in different biological processes may be used to reduce vector infestations and pathogen infection in ticks feeding on immunized animals (de la Fuente and Contreras, 2015). One novel approach to control populations might be to target specific endosymbionts, which requires detailed knowledge of microbial communities and their impact on tick biology (Taylor et al., 2012). Finally, the surveillance of microbial populations in tick salivary glands may enable the early identification of pathogens likely to be transmitted to vertebrate host (Qiu et al., 2014). Overall, the combination of effective and early diagnostics along with tick vaccines and strategies such as harnessing genetics to improve livestock breeds, and the rational application of acaricides, antivirals and other therapeutic interventions will result in a more effective and environmentally friendly control of tick populations. In addition, transgenic or paratransgenic ticks and vertebrate host genetically modified to confer resistance to pathogen infection may be produced and combined with vaccine applications and other interventions (de la Fuente and Kocan, 2014).

## Author contributions

JF, SA, SB, AD, AE, NJ, KM, AN, AP, NR, AF, ROMR conducted the literature research and wrote the paper. JF, AC, AP, SB, AN, NJ prepared the figures and tables. All authors provided critical review and revisions.

## Funding

Part of the research included in this review was supported by the Ministerio de Economia y Competitividad (Spain) grant BFU2016-79892-P and the European Union (EU) Seventh Framework Programme (FP7) ANTIGONE project number 278976. SA and AD would like to acknowledge FCT for funds to GHTM - UID/Multi/04413/2013. MV was supported by the Research Plan of the University of Castilla-La Mancha (UCLM), Spain. The funders had no role in study design, data collection and interpretation, or the decision to submit the work for publication.

### Conflict of interest statement

The authors declare that the research was conducted in the absence of any commercial or financial relationships that could be construed as a potential conflict of interest.
